# Dysfunction in the Cystic Fibrosis Transmembrane Regulator in Chronic Obstructive Pulmonary Disease as a Potential Target for Personalised Medicine

**DOI:** 10.3390/biomedicines9101437

**Published:** 2021-10-10

**Authors:** Laura Carrasco-Hernández, Esther Quintana-Gallego, Carmen Calero, Rocío Reinoso-Arija, Borja Ruiz-Duque, José Luis López-Campos

**Affiliations:** 1 Unidad Médico-Quirúrgica de Enfermedades Respiratorias, Instituto de Biomedicina de Sevilla (IBiS), Hospital Universitario Virgen del Rocío/Universidad de Sevilla, 41013 Sevilla, Spain; lauracarrascohdez@gmail.com (L.C.-H.); esther.quintana@telefonica.net (E.Q.-G.); ccalero-ibis@us.es (C.C.); rocioreari@gmail.com (R.R.-A.); borja_994@hotmail.com (B.R.-D.); 2 Centro de Investigación Biomédica en Red de Enfermedades Respiratorias (CIBERES), Instituto de Salud Carlos III, 28029 Madrid, Spain

**Keywords:** cystic fibrosis transmembrane conductance regulator, COPD, CFTR modulators, ivacaftor, icenticaftor

## Abstract

In recent years, numerous pathways were explored in the pathogenesis of COPD in the quest for new potential therapeutic targets for more personalised medical care. In this context, the study of the cystic fibrosis transmembrane conductance regulator (CFTR) began to gain importance, especially since the advent of the new CFTR modulators which had the potential to correct this protein’s dysfunction in COPD. The CFTR is an ion transporter that regulates the hydration and viscosity of mucous secretions in the airway. Therefore, its abnormal function favours the accumulation of thicker and more viscous secretions, reduces the periciliary layer and mucociliary clearance, and produces inflammation in the airway, as a consequence of a bronchial infection by both bacteria and viruses. Identifying CFTR dysfunction in the context of COPD pathogenesis is key to fully understanding its role in the complex pathophysiology of COPD and the potential of the different therapeutic approaches proposed to overcome this dysfunction. In particular, the potential of the rehydration of mucus and the role of antioxidants and phosphodiesterase inhibitors should be discussed. Additionally, the modulatory drugs which enhance or restore decreased levels of the protein CFTR were recently described. In particular, two CFTR potentiators, ivacaftor and icenticaftor, were explored in COPD. The present review updated the pathophysiology of the complex role of CFTR in COPD and the therapeutic options which could be explored.

## 1. Introduction

Despite the considerable advances made in recent years, the mechanisms underlying the onset, pathogenesis and symptomatic development of chronic obstructive pulmonary disease (COPD) remain largely unknown. Although we know that prolonged exposure to tobacco smoke and other inhaled toxins (e.g., biomass [1], and occupational smokes [2]) is the main risk factor for the disease, not all patients exposed to tobacco smoke develop this clinical condition. Furthermore, even among those who do develop COPD, the clinical, functional and prognostic impact varies among patients and the conditioning factors of this different evolution are equally unknown [3,4]. In this context, the search for pathogenetic pathways that help us understand the biological pathways that cause COPD, and which determine its clinical impact, constitute the current challenges in the biomedical research of this disease [5].

In recent decades, numerous pathways were explored that we now know play an important role in the pathogenesis of COPD, including protease–antiprotease imbalance, oxidative and nitrosative stress, inflammatory mechanisms associated with alterations in innate and acquired immunity, and apoptosis or autoimmunity phenomena [6]. However, despite all these efforts, the factor which defines the patients who will develop COPD when exposed to tobacco still eludes us. For this reason, a global initiative began to search for new frontiers of biological behaviour in COPD that could enable us to answer this question and, consequently, identify new therapeutic targets.

In this context, the study of the cystic fibrosis transmembrane conductance regulator (CFTR) started to gain importance in recent decades [7]. This interest heightened recently with the appearance of new drugs with the potential effect of modulating the physiology of this protein and having a potential impact on COPD [8]. The mucosal clearance from the airway is one of the main defence mechanisms of the airway. Bronchial mucus is capable of trapping foreign bodies due to its composition of water, mucins and salts, and it is continually carried into the upper airway by ciliary movement and the cough reflex. Therefore, this physiological function depends on the integrity of the cilia, the preservation of the cough reflex and the correct composition of the bronchial mucus. CFTR is a chlorine channel regulated by the cyclic adenosine monophosphate (cAMP) which is located in the apical membrane of bronchial epithelial cell and contributes to the movement of salts and water in the bronchial lumen, ensuring the correct composition and physiological behaviour of the mucus [9]. Alterations in the functioning of this protein lead to no water being secreted into the bronchial mucus, transforming it into a dehydrated mucus, which is more viscous and, therefore, more resistant to the movement of the cilia and their physiological function, thus weakening this defence mechanism of the respiratory system. This pathological condition is clearly seen in cystic fibrosis (CF) where there may be a complete absence of CFTR function [10]. In COPD, it is shown that a functional alteration of the CFTR contributes to its pathogenesis [7]. During this review, we aim to report the latest updates on the pathophysiology of CFTR in COPD and its possible treatments.

## 2. CFTR: Structure and Function

The gene that codes for this protein is located on the long arm of chromosome 7 and is made up of a 230 kb genomic sequence organized into 27 exons of different sizes [11]. The complementary deoxyribonucleic acid identifies a 6.1 kb transcript with 4400 nucleotides which encodes a protein with 1480 amino acids and a molecular weight of 170 kDa, known as the CFTR protein. This term was coined when it was recognized as the protein responsible for the appearance of CF, a genetic disease of autosomal recessive inheritance that originates from the failure of this protein [12].

The CFTR protein belongs to the family of adenosine triphosphate (ATP)-Binding Cassette transporters regulated by cAMP [13]. Structurally, it is made up of five domains: two transmembrane domains, made up of six segments each; a regulatory domain and two ATP binding domains, the so-called Nucleotide Binding Domain (NBD) 1 and 2. The NBD domains, the regulatory domain, and the NH2- and COOH-termini are found in the cytosol.

The activation of the CFTR protein requires a complex regulation involving the phosphorylation of the regulatory domain by protein kinase A and the subsequent binding and hydrolysis of ATP in the NBD domains. The binding of ATP with the cytosolic domain NBD1 produces the hydrolysis of ATP leading to the initiation of the opening of the channel. When ATP binding occurs with the NBD2 domain, the open channel is stabilised. Finally, the hydrolysis of ATP in NBD2 leads to the closure of the channel. If the regulatory domain remains phosphorylated, ATP binding and hydrolysis cycles occur and the channel opens and closes regularly [13].

CFTR is expressed in the epithelium of many exocrine organs, including the airway, lung, pancreas, liver, intestine, vas deferens and sweat glands. The protein acts as a chlorine channel and secretes chlorine, a process that contributes to the hydration of the airway, in addition to transporting bicarbonate and glutathione. It also plays an important role in regulating other membrane proteins, such as the sodium channel, whose key function, apart from CFTR, is homeostasis, controlling the movement of water and fluidising secretions in many organs [14]. Therefore, any alteration that occurs from the coding sequence to the synthesis of the mRNA or in other regions will give rise to an abnormal protein and, in turn, an alteration of its usual function.

## 3. CFTR Dysfunction in COPD

The acquired CFTR alteration in COPD is well described [15]. Briefly, the association of COPD with CFTR dysfunction relies on three main associations: the direct implication of tobacco smoke and the relationship between oxidative stress and CFTR physiology, as well as CFTR mutations.

### 3.1. CFTR and Tobacco Smoke

**Figure 1:**
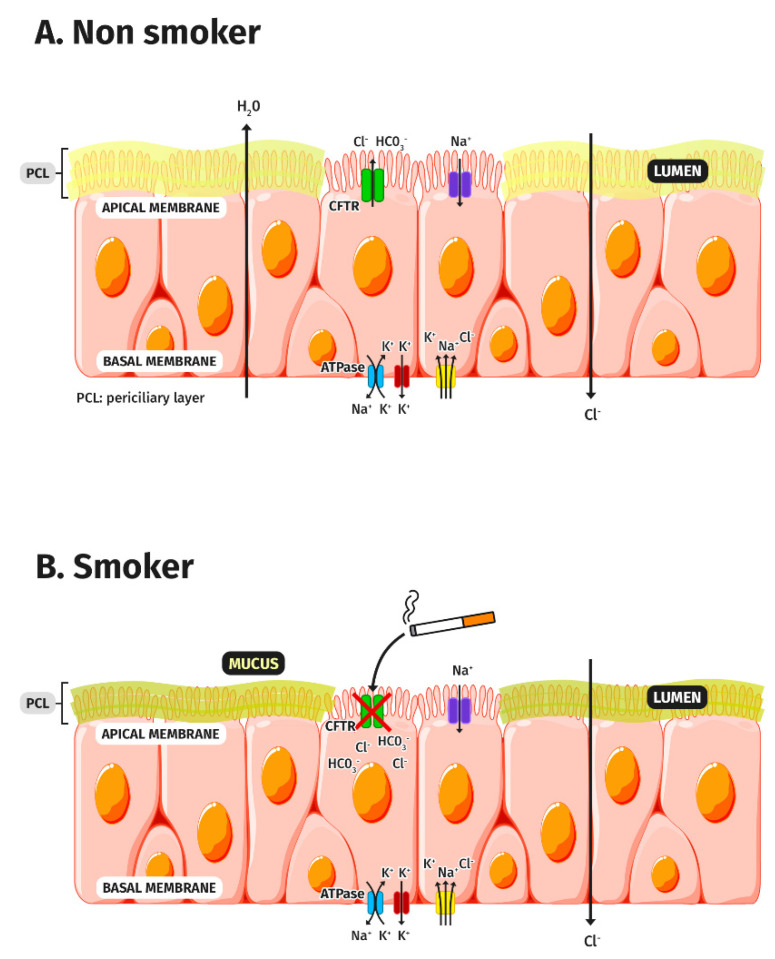
Model of airway surface dehydration in COPD due to CFTR dysfunction. (**A**) In non-smokers, an adequate exchange of ions occurs due to the correct functioning of the CFTR protein, located in the apical membrane of the respiratory epithelium. (**B**) In smokers, cigarette smoke produces a dysfunction of the CFTR protein producing an alteration of ion transport, making the mucus dehydrated, reducing the periciliary layer, and therefore hindering the expulsion of secretions.

The first identification of an acquired dysfunction of the CFTR by tobacco smoke was described in 1983 [16] (Figure 1). Since then, the impact of tobacco smoke on CFTR was seen in acute and chronic exposures and was demonstrated in in vivo and in vitro models. Several subsequent studies verified this acquired dysfunction and attempted to find its associated factors. Several actions are suggested for this phenomenon. First, several authors state that tobacco smoke is associated with the internalisation of the CFTR [17,18]. It seems that the increase in cytosolic free calcium, together with the exposure to tobacco smoke, is associated with a reduction in the cellular expression of CFTR, reducing the liquid secreted to the cell surface [19]. Additionally, an accelerated degradation of the CFTR is also described. Tobacco smoke can alter CFTR traffic by inducing internalization through the acute misfolding on the cell surface which causes it to disappear from this location, forming intracytoplasmic aggregates in the epithelial cells [17,18,20]. Finally, it is possible to show an alteration in the opening of the channel, which prevents its physiological functioning and increases the dehydration of the mucus. Therefore, three mechanisms are involved in CFTR COPD dysfunction: the reduced expression of the CFTR transcript, accelerated CFTR degradation (reduced stability), and altered channel gating.

Interestingly, this alteration of the CFTR has important connotations if we view it in the context with the remaining pathogenesis of COPD, such as the metaplasia and hyperplasia of goblet cells. The hypertrophy of the submucosal glands causes a state of hypersecretion in an altered mucus, leading to a reduced CFTR-mediated chlorine secretion and further airway mucus dehydration [21] which closes a dangerous vicious circle. Notably, this tobacco-induced CFTR dysfunction is also shown outside the lung in a manner analogous to CF, and is associated with pancreatic involvement and cachexia, suggesting that there could be a systemic effect due to a less well-known mediator [22].

Apart from the oxidative stress released by tobacco smoke, as discussed below, at least three main constituents of tobacco are directly associated with CFTR dysfunction: acrolein, ceramide and cadmium. Acrolein is a highly reactive metabolite of cigarette smoke that forms covalent bonds with various proteins and DNA [23]. In particular, acrolein can alter the CFTR by altering the opening of the channel [24]. Cadmium is a component of tobacco and an environmental pollutant that decreases CFTR expression and chlorine transport in in vitro models and human lungs [25]. Ceramides belong to a family of waxy lipid molecules composed of sphingosine and a fatty acid and are found in high concentrations within the cell membrane of the eukaryotic cells. In addition to their role as supporting structural elements, ceramides participate in a variety of cellular signals such as the regulation of cell differentiation and proliferation, as well as the apoptosis phenomena [26]. Exposure to cigarette smoke increases lung ceramide biosynthesis and alters its metabolic function. A number of recent studies demonstrated that the accumulation of ceramides associated with the exposure to tobacco smoke was related to the inhibition of CFTR expression [27].

### 3.2. CFTR and Oxidative Stress

Among the pathogenetic mechanisms involved in the genesis of COPD, oxidative stress is most likely plays a key role. It is important to bear in mind that the oxidation–antioxidation relationship maintains a delicate physiological balance in humans with a slight imbalance towards oxidative stress [28]. This physiological oxidative influx has important physiological functions which are necessary for the normal functioning of the various organs and systems. Therefore, it is termed oxidative stress when there is a greater imbalance in favour of the oxidative component of the balance [29]. This decompensation can occur either due to an increase in the oxidative influx or by a decrease in protective factors, such as enzymatic defences, including superoxide dismutase, catalase or the glutathione pathway.

In this regard, the relationship between CFTR dysfunction and oxidative stress seems to be the most important factor. The importance of the oxidative influence is shown as a factor that modulates the stability, physiology and expression of CFTR. In a recent study conducted with a mouse model, the authors described the lentivirus-mediated overexpression of CFTR. Compared to a negative control group, this overexpression of CFTR resulted in reduced levels of glutathione, reactive oxygen species, and malondialdehyde, together with an increase in superoxide dismutase, glutathione peroxidase, and total antioxidant capacity [30]. 

This CFTR-oxidative stress relationship is complex and most likely has a double significance. On the one hand, oxidative stress is reported to alter CFTR expression [31], Various studies show that oxidants related to cigarette smoke affect the expression and function of CFTR in the respiratory tract epithelia [31,32]. The mechanisms responsible for this effect are varied and involve different levels, including the reduced expression of CFTR transcription, the accelerated degradation of the protein and the alteration of the opening of the channel [17,33,34]. On the other hand, in cases of severe CFTR dysfunction, such as CF, an oxidative imbalance is described as leading to the increased production of reactive oxygen species [35]. If this double effect was demonstrated in COPD, this would lead to the consideration that oxidative stress not only played a central role in the pathogenesis, but had a clear therapeutic objective with which to break this possible redundant mechanism. To complete the picture, we need to bear in mind that the pathogenesis of COPD involves the generation of internal oxidative stress based on two other endogenous sources apart from tobacco smoke: hypoxia and chronic inflammation [36]. As a result, it is proposed that oxidative stress alone may be one of the most important factors in CFTR gene expression, density and physiology [31].

### 3.3. CFTR Mutations

**Figure 2:**
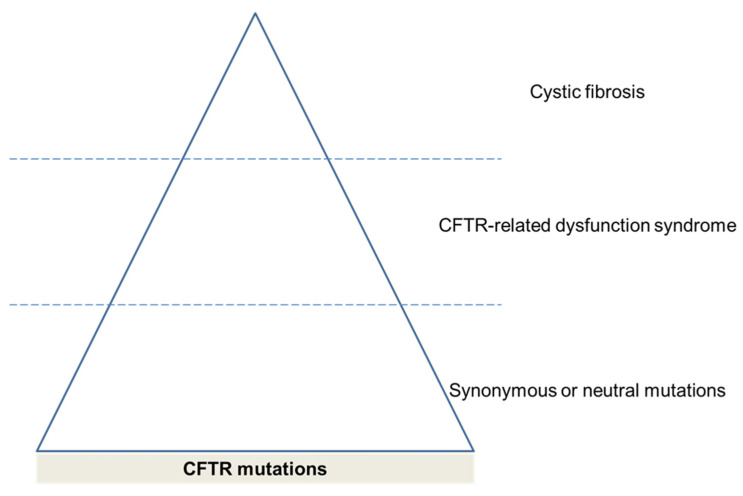
Classification of diseases as CFTR-related disorders.

The analysis of CFTR mutations and respiratory pathology needs some clarification. CF is an autosomal, recessive, inherited, genetic disease caused by mutations in the gene that encodes the CFTR protein. However, there are currently more than 1500 genetic variations of this gene which have a variable penetrance. In this way, the fact that mutations of uncertain significance are described as part of the CF diagnosis is currently under debate due to the low benefit derived from the diagnosis and the greater stress caused to the families. Accordingly, the term, CFTR-related dysfunction syndrome, was coined to identify these cases with a mutation of unknown clinical significance [37]. In addition, there were single-nucleotide polymorphisms which represented a change in one single nucleotide but had no associations with CF clinical presentations which might modify CFTR function. Therefore, the different CFTR mutations represent a spectrum of affections which start from the normal protein expression and move to a frank deficit resulting in CF (Figure 2).

CFTR mutations in patients with chronic airway diseases such as bronchiectasis and COPD are previously described [38]. Although not many variants are associated with any disorder so far, different alleles are found to be more frequent in COPD patients [39]. Accordingly, although these alterations occur in the absence of a CF-associated mutation (since in these cases a diagnosis of CF should be considered as an alternative), the genetic variations of CFTR associated with COPD are also described [39,40,41]. 

The allele present in the polymorphic locus, M470V (1540A = G in exon 10), affected the biogenesis and the gating of the CFTR channel. The M470 CFTR proteins had a 1.7-fold increase in intrinsic chloride activity compared with the V470 protein [39]. This hyperactive M470 variant was found more frequently in COPD patients than in the controls. In particular, the VV470 genotypes saw a 3.4-fold decrease in the risk of the appearance of severe/very severe COPD symptoms, which suggested that they played a protective role. Interestingly, it was suggested that the combination of certain alleles at various loci could result in a dysfunctional CFTR protein to different degrees [42]. Consequently, the combination with other mutations could affect the final phenotypic expression producing non-functional CFTR proteins which affected the net activity of chlorine transport [39,41]. Similarly, the R75Q polymorphism (356G = A in exon 3) was associated with COPD [40]. Of note, the patients who were homozygous for the V470 allele carrying the R75Q variant had chronic bronchitis as the dominant symptom of COPD, which suggested a link with CFTR dysfunction [40].

## 4. Consequences of CFTR Dysfunction in COPD

There are two main controversies regarding the relationship between CFTR dysfunction and COPD. The first is the role of CFTR dysfunction as a risk factor for COPD. Here, the evidence of a tobacco smoke-associated CFTR dysfunction seems clear. However, if CFTR plays a key role in the development of COPD, a more impaired CFTR function in COPD should be expected when compared to healthy smokers. However, this seems not to be the case in the available studies which show a similar degree of impairment [21,43]. Interestingly, there are clinical data suggesting this association. From a biological perspective, at the macrophage level, CFTR dysfunction is related to the impaired bacterial phagocytosis and favours recurrent bacterial infections and airway inflammation [44], one key finding of COPD pathogenesis. From a clinical perspective, two studies show some relationship between COPD and CFTR dysfunction. A study comparing healthy non-smokers with current smokers, with and without COPD, showed a significant reduction in CFTR activity with correlations of this activity with the presence of chronic bronchitis and dyspnoea scores [21]. In patients with emphysema, it was shown that the amount of CFTR in the lung tissue was directly related to the spirometric values of lung function, in both the forced vital capacity and the forced expiratory volume in one second (FEV_1_), and inversely related to the stages of the functional involvement of COPD [45].

The second source of debate is the role of CFTR dysfunction in the different COPD clinical phenotypes: chronic bronchitis and emphysema. The consequences of CFTR dysfunction in COPD are described in both anatomical compartments related to the disease: the airway and the lung parenchyma. On the one hand, one direct consequence of having a thicker mucus implies that the cilia cannot easily remove it. As a consequence, the cough mechanism increases to try to compensate for this deficit, with an increase in coughing and chronic expectoration, which is called chronic bronchitis [46]. On the other hand, as mentioned above, in patients with emphysema, there is a correlation between the spirometric values of lung function and CFTR [45], Interestingly, a recent murine model shows the contribution of a reduced expression of CFTR and airspace enlargement [47].

Altogether, the evidence suggests that smokers with and without COPD have a reduced lower airway CFTR activity compared with healthy non-smokers, and this finding correlates with the clinical expression of COPD. Notably, there are still dark spots in the relationship between COPD and CFTR. In this context, the development of animal models could clarify the relationship between CFTR, COPD and modulatory treatments. A possible initiative would be the study of CFTR-deficient mice exposed to smoke-induced COPD mouse model, not yet developed. Additionally, the response to treatments which influence CFTR function or its consequences could shed some light on this debate.

## 5. Treatments for Improving CFTR Function in COPD

**Figure 3:**
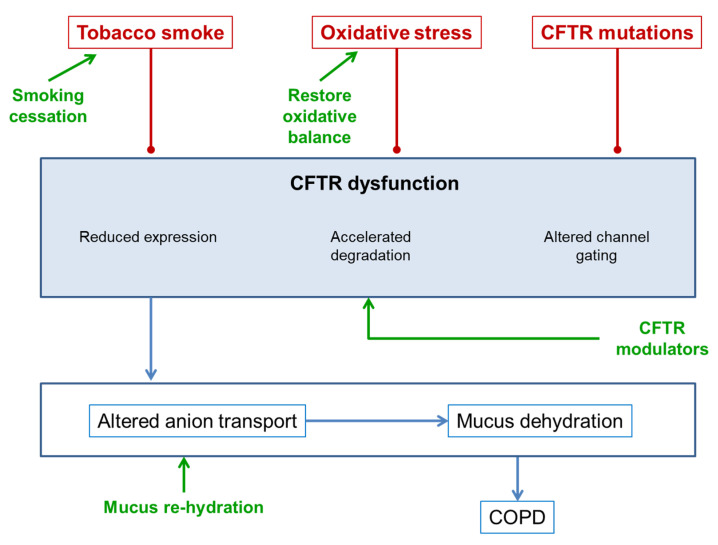
Therapeutic points of action of CFTR dysfunction.

Consequently, CTFR dysfunction, either innately due to a genetic alteration or by acquiring tobacco smoke and oxidative stress, is described in both diseases, CF and COPD. Therefore, it could be suggested that treatments to improve CFTR function in CF could be applicable to COPD (Figure 3). In particular, the fact that both diseases share pathophysiological mechanisms and clinical expressions, such as airway inflammation, goblet cell metaplasia, a reduced mucociliar clearance, mucus hypersecretion, small airways’ mucus obstruction, and chronic bacterial infections, in addition to the importance of CFTR dysfunction in COPD mentioned above, makes it possible to consider the option of common treatments for both processes.

### 5.1. Smoking Cessation

CFTR dysfunction due to exposure to tobacco smoke is partially reversible after smoking cessation, which justifies a cause-and-effect relationship between exposure to tobacco smoke and CFRT dysfunction [17,18]. However, it is important to bear in mind that the inflammation in COPD and its pathophysiological mechanisms are perpetuated with the severity of the lung disease [48,49]. Therefore, it is likely that, once established, the mechanisms other than the direct exposure to tobacco smoke contribute to maintaining an altered function of the CFTR. In any case, all initiatives that help COPD patients to quit smoking should be prioritized in the healthcare of these patients [50,51].

### 5.2. Rehydration of Mucus

Since CFTR dysfunction leads to the dehydration of the mucus, one key therapeutic target would be the rehydration of the mucus, since this would improve mucociliary clearance, and therefore reduce the obstruction produced by this mucus. The administration of a hypertonic serum spray is shown to restore mucus hydration, increase periciliary fluid volume and improve bronchial clearance [52]. A study with models of dehydrated cells shows that the application of hypertonic saline is able to restore the height of the mucus by improving its hydration [17]. Unfortunately, however, this effect seems to be short-lived. Another option would be the administration of a hypertonic saline solution, which results in a higher concentration of salt on the surfaces of the airways [53]. This increase in salts generates a chlorine gradient that is absorbed through the partially functional channel mediated by CFTR and the paracellular pathway, together with the absorption of sodium by the epithelial sodium channel [54]. This resulting transfer of salt limits the duration of the serum’s osmotic effects. Therefore, it is likely that frequent doses of hypertonic solution would be needed. This effect, however, does not occur in CF, where the CFTR is not functioning or is completely absent, so that the passage of chlorine, though it is inhibited, limits sodium absorption, and therefore results in a better-maintained osmotic effect [55].

The inhibition of the epithelial sodium channel is tested in normal human bronchial epithelial cultures, which suggests a possible associative mechanism to improve the efficacy of this therapeutic option [46]. Here, the inhibition of the epithelial sodium channel improves hydration in patients with impaired CFTR and the search for epithelial sodium channel inhibitors as possible treatments is beginning [52]. However, the clinical effects of this therapeutic strategy did not yield sufficient results [56,57] and new approaches are being sought. For example, ursodeoxycholic acid has immunomodulatory and epithelial ion transport-enhancing properties. Recent work shows that this acid can inhibit the epithelial sodium channel activity by improving hydration in a model of normal human airway epithelial cells and cystic fibrosis, suggesting the therapeutic potential for ursodeoxycholic acid in CF lung disease [58].

### 5.3. Antioxidants

Due to the pathophysiology outlined above, counteracting oxidative stress could be another strategy worth exploring. Here, scavengers were tested to see if they could at least partially reduce the function of CFTR, and therefore could be used in the treatment of COPD [59]. In addition, nitric oxide and S-nitrosoglutathione play a crucial role in maintaining functional lung homeostasis under physiological conditions, in which intracellular levels of S-nitrosoglutathione are controlled by the S-nitrosoglutathione reductase enzyme that degrades S-nitrosoglutathione [60]. Some authors show how increasing the S-nitrosoglutathione levels improves the pathogenesis of COPD by reducing the acquired CFTR dysfunction [61]. In a murine animal model, some authors show that this treatment leads to an improvement by increasing the autophagy phenomena [62], which suggests a relevant role of this autophagy in the pathogenesis of COPD and its relationship with CFTR dysfunction. Therefore, increasing S-nitrosoglutathione levels could be a promising strategy to treat COPD due to the ability of S-nitrosoglutathione to increase CFTR expression and maturation [63]. In fact, the therapeutic benefits of the inhibitors of S-nitrosoglutathione reductase (N6022) are already tested [61]. These authors used biobanked paraffin-embedded lung tissue sections, a murine model with C57BL/6 mice and Beas2b cells cultures to evaluate the effects of S-nitrosoglutathione augmentation in regulating inflammatory-oxidative stress and COPD-emphysema pathogenesis. Altogether, the authors conclude that augmenting S-nitrosoglutathione levels controls COPD-emphysema pathogenesis by reducing cigarette smoke-induced acquired CFTR dysfunction and resulting in autophagy impairment and chronic inflammatory–oxidative stress.

### 5.4. Phosphodiesterase Inhibitors

The intracellular levels of cAMP are another interesting therapeutic target, due to the important role of cAMP in the physiology of CFTR [64]. The role of cAMP in COPD is studied both in the intracellular pathways that mediate inflammation and in the physiological and pharmacological bronchodilator response. In this context, phosphodiesterases (PDE) can break down cAMP and regulate the intracellular concentrations of cAMP. As a consequence, PDE inhibitors can prevent cAMP degradation and consequently restore CFTR function. PDE constitute a large family of inhibitors from which 11 types are known in humans [65]. Ubiquitously located, PDE3 and PDE4 seem to play a relevant role in the respiratory system. So far, we have a non-selective inhibitor of PDE such as xanthines. In addition, we currently have a selective PDE4 inhibitor, roflumilast [66], and a dual PDE3/4 inhibitor in development that has anti-inflammatory and bronchodilator effects [67]. The role of roflumilast in the treatment of COPD is well established in current guidelines for the management of the disease [4] and dual PDE3/4 inhibitors are under development [67].

Recently, several preclinical studies showed that roflumilast could benefit COPD patients with chronic bronchitis by activating CFTR and restoring its function [68,69]. This effect on CFTR activity was also demonstrated in animal models [70]. In addition to its ability to partially restore tobacco-induced CFTR dysfunction in bronchial epithelial cells, roflumilast combined with adenosine increased mucosal hydration in human airway epithelial cultures after cigarette smoke exposure [71].

## 6. CFTR Modulators

Today, there is a new generation of drugs available known as CFTR modulator drugs [72,73], which are small molecules which enhance CFTR or restore the decreased levels of proteins on the cell surface. These drugs were initially synthesized to correct the CFTR genetic defects that occurred in CF. However, attempts are now being made to provide the drug with another function, that is, in acquired CFTR dysfunction, such as in COPD. There are three main types of CFTR modulators: CFTR potentiators (ivacaftor and icenticaftor) hold the protein gate open so chloride can flow through the cell membrane; CFTR correctors (lumacaftor, tezacaftor, and elexacaftor) help the CFTR protein to form the right 3-D shape so that it is able to move, or traffic, to the cell surface; and CFTR amplifiers (under development) increase the amount of CFTR protein that the cell produces. Currently, the therapeutic strategy for CF includes the combination of several of these molecules to increase therapeutic efficacy and tolerability. To date, only ivacaftor and, more recently, icenticaftor are explored in COPD.

### 6.1. Ivacaftor and COPD

Ivacaftor (VX-770) seems to play a role as a CFTR potentiator in diseases that present with the acquired CFTR dysfunction. Ivacaftor is shown to reverse the changes made by tobacco smoke in the human bronchial epithelium in cell cultures by increasing the probability of channel opening, improving the chlorine conductance, restoring cell surface fluid and improving mucociliary clearance [68,74,75]. Although clinical trials of CFTR-enhancing drugs in COPD patients are in the early stages, a recent study shows that ivacaftor in patients with chronic bronchitis leads to an improvement in symptoms and chlorine levels in the sweat test [76]. Currently, a Phase 2 clinical trial (the TOPIC trial), aiming to establish the safety and efficacy of ivacaftor in COPD patients with chronic bronchitis and acquired CFTR dysfunction as detected by sweat chloride analysis, is recruiting patients (ClinicalTrials.gov Identifier: NCT03085485 (accessed on 30 July 2021)). The design is a pilot, randomized (3:1, active:placebo), double-blind, placebo-controlled study, and approximately 40 subjects with COPD will be randomized.

### 6.2. Icenticaftor and COPD

Icenticaftor (QBW251) is a CFTR potentiator molecule that can restore CFTR dysfunction in certain CF genotypes [77]. A study on the efficacy and safety of Icenticaftor in COPD patients was recently published [8]. This multicentre, randomized, double-blind, placebo-controlled study included 92 patients with moderate/severe COPD. The study consisted of 2 weeks when the patients were treated with a placebo, to verify the stability of the baseline treatment of COPD, followed by a period of 4 weeks where the patients took the placebo twice a day or icenticaftor 300 mg twice a day, followed by a final 4 weeks of single-blind placebo. The primary endpoint was the change from the baseline to day 29 in the lung clearance index of icenticaftor vs. placebo. The secondary objective was to compare the changes between the baseline and day 29 of prebronchodilation and postbronchodilation FEV_1_. Other endpoints studied were the changes in the sweat test, plasma fibrinogen levels and sputum colonization.

The results showed that, by day 29, icenticaftor did not improve the change in the lung clearance index (treatment difference: 0.28, with a 19% probability of being more effective than the placebo), but did show an improvement in prebronchodilator FEV_1_ (mean: 50 mL with an 84% probability of being more effective) and in postbronchodilator FEV_1_ (mean: 63 mL, with a 91% probability of being more effective than the placebo).

Improvements were also observed in the bacterial colonization, sweat test results, fibrinogen in plasma and bacterial colonization of sputum. Regarding safety, the drug was shown to be both safe and well-tolerated [8].

## 7. Conclusions

CFTR dysfunction is an area of the pathophysiology of COPD which offers opportunities for new therapeutic targets and a more personalised approach. Understanding its underlying biological pathways may help us to identify the novel initiatives which may lead to valid therapeutic options for specific patient types. Due to the fact that the clinical features of these patients were similar to those observed in the CF patients, with a chronic cough and expectoration leading to thicker and more viscous secretions, the option of being able to use CFTR modulating drugs in COPD is now being explored.

## References

[1] Capistrano S.J., van Reyk D., Chen H., Oliver B.G. (2017). Evidence of biomass smoke exposure as a causative factor for the development of copd. Toxics.

[2] Kraim-Leleu M., Lesage F.X., Drame M., Lebargy F., Deschamps F. (2016). Occupational risk factors for copd: A case-control study. PLoS ONE.

[3] López-Campos J.L., Calle Rubio M., Izquierdo Alonso J.L., Fernández-Villar A., Abascal-Bolado B., Alcázar B., García-Río F., Peces-Barba G., Serra Batlles J., Martínez Garcerán J.J. (2021). Forum copd working group consensus on the diagnosis, treatment and follow-up of copd. Arch. Bronconeumol..

[4] Miravitlles M., Calle M., Molina J., Almagro P., Gomez J.T., Trigueros J.A., Cosio B.G., Casanova C., Lopez-Campos J.L., Riesco J.A. (2021). Spanish copd guidelines (gesepoc) 2021: Updated pharmacological treatment of stable copd. Arch. Bronconeumol..

[5] Celli B.R., Decramer M., Wedzicha J.A., Wilson K.C., Agusti A.A., Criner G.J., MacNee W., Make B.J., Rennard S.I., Stockley R.A. (2015). An official american thoracic society/european respiratory society statement: Research questions in copd. Eur. Respir. Rev..

[6] Agusti A., Faner R. (2020). Chronic obstructive pulmonary disease pathogenesis. Clin. Chest Med..

[7] Fernandez Fernandez E., De Santi C., De Rose V., Greene C.M. (2018). CFTR dysfunction in cystic fibrosis and chronic obstructive pulmonary disease. Expert Rev. Respir. Med..

[8] Rowe S.M., Jones I., Dransfield M.T., Haque N., Gleason S., Hayes K.A., Kulmatycki K., Yates D.P., Danahay H., Gosling M. (2020). Efficacy and safety of the CFTR potentiator icenticaftor (qbw251) in copd: Results from a phase 2 randomized trial. Int. J. Chron. Obstruct. Pulmon. Dis..

[9] Kunzelmann K., Mehta A. (2013). CFTR: A hub for kinases and crosstalk of cAMP and Ca^2+^. FEBS J..

[10] Cantin A.M., Hartl D., Konstan M.W., Chmiel J.F. (2015). Inflammation in cystic fibrosis lung disease: Pathogenesis and therapy. J. Cyst. Fibros..

[11] Riordan J.R., Rommens J.M., Kerem B., Alon N., Rozmahel R., Grzelczak Z., Zielenski J., Lok S., Plavsic N., Chou J.L. (1989). Identification of the cystic fibrosis gene: Cloning and characterization of complementary DNA. Science.

[12] Mingora C.M., Flume P.A. (2021). Pulmonary complications in cystic fibrosis: Past, present and future. Chest.

[13] Csanády L., Vergani P., Gadsby D.C. (2019). Structure, gating, and regulation of the CFTR anion channel. Physiol. Rev..

[14] Stutts M.J., Canessa C.M., Olsen J.C., Hamrick M., Cohn J.A., Rossier B.C., Boucher R.C. (1995). CFTR as a cAMP-dependent regulator of sodium channels. Science.

[15] Shi J., Li H., Yuan C., Luo M., Wei J., Liu X. (2018). Cigarette smoke-induced acquired dysfunction of cystic fibrosis transmembrane conductance regulator in the pathogenesis of chronic obstructive pulmonary disease. Oxid Med. Cell. Longev..

[16] Welsh M.J. (1983). Cigarette smoke inhibition of ion transport in canine tracheal epithelium. J. Clin. Investig..

[17] Clunes L.A., Davies C.M., Coakley R.D., Aleksandrov A.A., Henderson A.G., Zeman K.L., Worthington E.N., Gentzsch M., Kreda S.M., Cholon D. (2012). Cigarette smoke exposure induces CFTR internalization and insolubility, leading to airway surface liquid dehydration. FASEB J..

[18] Marklew A.J., Patel W., Moore P.J., Tan C.D., Smith A.J., Sassano M.F., Gray M.A., Tarran R. (2019). Cigarette smoke exposure induces retrograde trafficking of CFTR to the endoplasmic reticulum. Sci. Rep..

[19] Rasmussen J.E., Sheridan J.T., Polk W., Davies C.M., Tarran R. (2014). Cigarette smoke-induced Ca^2+^ release leads to cystic fibrosis transmembrane conductance regulator (CFTR) dysfunction. J. Biol. Chem..

[20] Younger J.M., Chen L., Ren H.Y., Rosser M.F., Turnbull E.L., Fan C.Y., Patterson C., Cyr D.M. (2006). Sequential quality-control checkpoints triage misfolded cystic fibrosis transmembrane conductance regulator. Cell.

[21] Dransfield M.T., Wilhelm A.M., Flanagan B., Courville C., Tidwell S.L., Raju S.V., Gaggar A., Steele C., Tang L.P., Liu B. (2013). Acquired cystic fibrosis transmembrane conductance regulator dysfunction in the lower airways in copd. Chest.

[22] Raju S.V., Jackson P.L., Courville C.A., McNicholas C.M., Sloane P.A., Sabbatini G., Tidwell S., Tang L.P., Liu B., Fortenberry J.A. (2013). Cigarette smoke induces systemic defects in cystic fibrosis transmembrane conductance regulator function. Am. J. Respir. Crit. Care Med..

[23] Stevens J.F., Maier C.S. (2008). Acrolein: Sources, metabolism, and biomolecular interactions relevant to human health and disease. Mol. Nutr. Food Res..

[24] Alexander N.S., Blount A., Zhang S., Skinner D., Hicks S.B., Chestnut M., Kebbel F.A., Sorscher E.J., Woodworth B.A. (2012). Cystic fibrosis transmembrane conductance regulator modulation by the tobacco smoke toxin acrolein. Laryngoscope.

[25] Hassan F., Xu X., Nuovo G., Killilea D.W., Tyrrell J., Da Tan C., Tarran R., Diaz P., Jee J., Knoell D. (2014). Accumulation of metals in gold4 copd lungs is associated with decreased CFTR levels. Respir. Res..

[26] Gomez-Larrauri A., Presa N., Dominguez-Herrera A., Ouro A., Trueba M., Gomez-Muñoz A. (2020). Role of bioactive sphingolipids in physiology and pathology. Essays Biochem..

[27] Bodas M., Min T., Mazur S., Vij N. (2011). Critical modifier role of membrane-cystic fibrosis transmembrane conductance regulator-dependent ceramide signaling in lung injury and emphysema. J. Immunol..

[28] Newsholme P., Cruzat V.F., Keane K.N., Carlessi R., de Bittencourt P.I. (2016). Molecular mechanisms of ros production and oxidative stress in diabetes. Biochem. J..

[29] Brieger K., Schiavone S., Miller F.J., Krause K.H. (2012). Reactive oxygen species: From health to disease. Swiss Med. Wkly..

[30] Xu X., Huang H., Yin X., Fang H., Shen X. (2020). Effect of lentivirus-mediated CFTR overexpression on oxidative stress injury and inflammatory response in the lung tissue of copd mouse model. Biosci. Rep..

[31] Cantin A.M., Bilodeau G., Ouellet C., Liao J., Hanrahan J.W. (2006). Oxidant stress suppresses CFTR expression. Am. J. Physiol. Cell Physiol..

[32] Zhang Z., Leir S.H., Harris A. (2015). Oxidative stress regulates CFTR gene expression in human airway epithelial cells through a distal antioxidant response element. Am. J. Respir. Cell Mol. Biol..

[33] Braun A.P. (2014). Cigarette smoke and calcium conspire to impair CFTR function in airway epithelia. Channels.

[34] O′Grady S.M. (2019). Oxidative stress, autophagy and airway ion transport. Am. J. Physiol. Cell Physiol..

[35] Kunzi L., Easter M., Hirsch M.J., Krick S. (2021). Cystic fibrosis lung disease in the aging population. Front. Pharmacol..

[36] Asensio-Cruz M.I., Calero-Acuña C., Arellano-Orden E., Sánchez-López V., Caballero-Eraso C., Cejudo P., Lopez-Villalobos J.L., Lopez-Campos J.L., Ortega-Ruiz F., Sánchez-Armengol Á. (2020). Differences in overexpression of hypoxia-induced transcription factors and associated biomarkers in three different types of chronic hypoxia. Arch. Bronconeumol..

[37] Bombieri C., Claustres M., De Boeck K., Derichs N., Dodge J., Girodon E., Sermet I., Schwarz M., Tzetis M., Wilschanski M. (2011). Recommendations for the classification of diseases as CFTR-related disorders. J. Cyst. Fibros..

[38] Pignatti P.F., Bombieri C., Marigo C., Benetazzo M., Luisetti M. (1995). Increased incidence of cystic fibrosis gene mutations in adults with disseminated bronchiectasis. Hum. Mol. Genet..

[39] Tzetis M., Efthymiadou A., Strofalis S., Psychou P., Dimakou A., Pouliou E., Doudounakis S., Kanavakis E. (2001). CFTR gene mutations--including three novel nucleotide substitutions--and haplotype background in patients with asthma, disseminated bronchiectasis and chronic obstructive pulmonary disease. Hum. Genet..

[40] Divac A., Nikolic A., Mitic-Milikic M., Nagorni-Obradovic L., Petrovic-Stanojevic N., Dopudja-Pantic V., Nadaskic R., Savic A., Radojkovic D. (2004). High frequency of the r75q CFTR variation in patients with chronic obstructive pulmonary disease. J. Cyst. Fibros..

[41] Stankovic M., Nikolic A., Divac A., Tomovic A., Petrovic-Stanojevic N., Andjelic M., Dopudja-Pantic V., Surlan M., Vujicic I., Ponomarev D. (2008). The CFTR m470v gene variant as a potential modifier of copd severity: Study of serbian population. Genet. Test..

[42] Cuppens H., Lin W., Jaspers M., Costes B., Teng H., Vankeerberghen A., Jorissen M., Droogmans G., Reynaert I., Goossens M. (1998). Polyvariant mutant cystic fibrosis transmembrane conductance regulator genes. The polymorphic (tg)m locus explains the partial penetrance of the t5 polymorphism as a disease mutation. J. Clin. Investig..

[43] Courville C.A., Tidwell S., Liu B., Accurso F.J., Dransfield M.T., Rowe S.M. (2014). Acquired defects in CFTR-dependent beta-adrenergic sweat secretion in chronic obstructive pulmonary disease. Respir. Res..

[44] Ni I., Ji C., Vij N. (2015). Second-hand cigarette smoke impairs bacterial phagocytosis in macrophages by modulating CFTR dependent lipid-rafts. PLoS ONE.

[45] Zhao R., Liang X., Zhao M., Liu S.L., Huang Y., Idell S., Li X., Ji H.L. (2014). Correlation of apical fluid-regulating channel proteins with lung function in human copd lungs. PLoS ONE.

[46] Åstrand A.B., Hemmerling M., Root J., Wingren C., Pesic J., Johansson E., Garland A.L., Ghosh A., Tarran R. (2015). Linking increased airway hydration, ciliary beating, and mucociliary clearance through enac inhibition. Am. J. Physiol. Lung Cell Mol. Physiol..

[47] Wellmerling J.H., Chang S.W., Kim E., Osman W.H., Boyaka P.N., Borchers M.T., Cormet-Boyaka E. (2019). Reduced expression of the ion channel CFTR contributes to airspace enlargement as a consequence of aging and in response to cigarette smoke in mice. Respir. Res..

[48] Li Q.Y., Huang S.G., Wan H.Y., Wu H.C., Zhou T., Li M., Deng W.W. (2007). Effect of smoking cessation on airway inflammation of rats with chronic bronchitis. Chin. Med. J..

[49] Rutgers S.R., Postma D.S., ten Hacken N.H., Kauffman H.F., van Der Mark T.W., Koeter G.H., Timens W. (2000). Ongoing airway inflammation in patients with copd who do not currently smoke. Thorax.

[50] López-Campos J.L., Jiménez-Ruiz C.A., Meneses Petersen E.D., Rabade Castedo C., Asensio Sánchez S., Vaquero Lozano P., Ferrer Espinosa S., Pérez Soriano M.D.P., de Higes Martínez E., García de Llanos C. (2021). Smoking cessation units as a source of copd diagnoses: Project 1000-200. Arch. Bronconeumol..

[51] Cabrera López C., Gómez Sáenz J.T., Molina París J., Trigueros Carrero J.A., López-Campos J.L. (2021). Enabling a community approach to respiratory diseases: The hacer copd project. Arch. Bronconeumol..

[52] Mall M.A. (2016). Unplugging mucus in cystic fibrosis and chronic obstructive pulmonary disease. Ann. Am. Thorac Soc..

[53] Daviskas E., Robinson M., Anderson S.D., Bye P.T. (2002). Osmotic stimuli increase clearance of mucus in patients with mucociliary dysfunction. J. Aerosol Med..

[54] Mall M.A., Hartl D. (2014). CFTR: Cystic fibrosis and beyond. Eur. Respir. J..

[55] Donaldson S.H., Bennett W.D., Zeman K.L., Knowles M.R., Tarran R., Boucher R.C. (2006). Mucus clearance and lung function in cystic fibrosis with hypertonic saline. N. Engl. J. Med..

[56] Decramer M., Rutten-van Molken M., Dekhuijzen P.N., Troosters T., van Herwaarden C., Pellegrino R., van Schayck C.P., Olivieri D., Del Donno M., De Backer W. (2005). Effects of n-acetylcysteine on outcomes in chronic obstructive pulmonary disease (bronchitis randomized on nac cost-utility study, broncus): A randomised placebo-controlled trial. Lancet.

[57] Zheng J.P., Kang J., Huang S.G., Chen P., Yao W.Z., Yang L., Bai C.X., Wang C.Z., Wang C., Chen B.Y. (2008). Effect of carbocisteine on acute exacerbation of chronic obstructive pulmonary disease (peace study): A randomised placebo-controlled study. Lancet.

[58] Mroz M.S., Harvey B.J. (2019). Ursodeoxycholic acid inhibits enac and na/k pump activity to restore airway surface liquid height in cystic fibrosis bronchial epithelial cells. Steroids.

[59] Vij N. (2017). Nano-based rescue of dysfunctional autophagy in chronic obstructive lung diseases. Expert Opin. Drug Deliv..

[60] Sun X., Qiu J., Strong S.A., Green L.S., Wasley J.W., Blonder J.P., Colagiovanni D.B., Mutka S.C., Stout A.M., Richards J.P. (2011). Discovery of potent and novel s-nitrosoglutathione reductase inhibitors devoid of cytochrome p450 activities. Bioorg. Med. Chem. Lett..

[61] Bodas M., Silverberg D., Walworth K., Brucia K., Vij N. (2017). Augmentation of s-nitrosoglutathione controls cigarette smoke-induced inflammatory-oxidative stress and chronic obstructive pulmonary disease-emphysema pathogenesis by restoring cystic fibrosis transmembrane conductance regulator function. Antioxid. Redox Signal..

[62] Bodas M., Pehote G., Silverberg D., Gulbins E., Vij N. (2019). Autophagy augmentation alleviates cigarette smoke-induced CFTR-dysfunction, ceramide-accumulation and copd-emphysema pathogenesis. Free Radic. Biol. Med..

[63] Zaman K., Sawczak V., Zaidi A., Butler M., Bennett D., Getsy P., Zeinomar M., Greenberg Z., Forbes M., Rehman S. (2016). Augmentation of CFTR maturation by s-nitrosoglutathione reductase. Am. J. Physiol. Lung Cell Mol. Physiol..

[64] Nguyen J.P., Bianca M., Huff R.D., Tiessen N., Inman M.D., Hirota J.A. (2021). Modulation of cAMP metabolism for CFTR potentiation in human airway epithelial cells. Sci. Rep..

[65] Padda I.S., Tripp J. (2021). Phosphodiesterase inhibitors. Statpearls.

[66] Lam J., Tonnu-Mihara I., Kenyon N.J., Kuhn B.T. (2021). Comparative effectiveness of roflumilast and azithromycin for the treatment of chronic obstructive pulmonary disease. Chronic Obstr. Pulm. Dis..

[67] Martin C., Burgel P.R., Roche N. (2021). Inhaled dual phosphodiesterase 3/4 inhibitors for the treatment of patients with copd: A short review. Int. J. Chron. Obstruct. Pulmon. Dis..

[68] Lambert J.A., Raju S.V., Tang L.P., McNicholas C.M., Li Y., Courville C.A., Farris R.F., Coricor G.E., Smoot L.H., Mazur M.M. (2014). Cystic fibrosis transmembrane conductance regulator activation by roflumilast contributes to therapeutic benefit in chronic bronchitis. Am. J. Respir. Cell Mol. Biol..

[69] Schmid A., Baumlin N., Ivonnet P., Dennis J.S., Campos M., Krick S., Salathe M. (2015). Roflumilast partially reverses smoke-induced mucociliary dysfunction. Respir. Res..

[70] Raju S.V., Rasmussen L., Sloane P.A., Tang L.P., Libby E.F., Rowe S.M. (2017). Roflumilast reverses CFTR-mediated ion transport dysfunction in cigarette smoke-exposed mice. Respir. Res..

[71] Tyrrell J., Qian X., Freire J., Tarran R. (2015). Roflumilast combined with adenosine increases mucosal hydration in human airway epithelial cultures after cigarette smoke exposure. Am. J. Physiol. Lung Cell Mol. Physiol..

[72] Patel S.D., Bono T.R., Rowe S.M., Solomon G.M. (2020). CFTR targeted therapies: Recent advances in cystic fibrosis and possibilities in other diseases of the airways. Eur. Respir. Rev..

[73] Spanò V., Venturini A., Genovese M., Barreca M., Raimondi M.V., Montalbano A., Galietta L.J.V., Barraja P. (2020). Current development of CFTR potentiators in the last decade. Eur. J. Med. Chem..

[74] Sloane P.A., Shastry S., Wilhelm A., Courville C., Tang L.P., Backer K., Levin E., Raju S.V., Li Y., Mazur M. (2012). A pharmacologic approach to acquired cystic fibrosis transmembrane conductance regulator dysfunction in smoking related lung disease. PLoS ONE.

[75] Raju S.V., Lin V.Y., Liu L., McNicholas C.M., Karki S., Sloane P.A., Tang L., Jackson P.L., Wang W., Wilson L. (2017). The cystic fibrosis transmembrane conductance regulator potentiator ivacaftor augments mucociliary clearance abrogating cystic fibrosis transmembrane conductance regulator inhibition by cigarette smoke. Am. J. Respir. Cell Mol. Biol..

[76] Solomon G.M., Hathorne H., Liu B., Raju S.V., Reeves G., Acosta E.P., Dransfield M.T., Rowe S.M. (2016). Pilot evaluation of ivacaftor for chronic bronchitis. Lancet Respir. Med..

[77] Grand D.L., Gosling M., Baettig U., Bahra P., Bala K., Brocklehurst C., Budd E., Butler R., Cheung A.K., Choudhury H. (2021). Discovery of icenticaftor (qbw251), a cystic fibrosis transmembrane conductance regulator potentiator with clinical efficacy in cystic fibrosis and chronic obstructive pulmonary disease. J. Med. Chem..

